# Surfactant Protein D Deficiency in Mice Is Associated with Hyperphagia, Altered Fat Deposition, Insulin Resistance, and Increased Basal Endotoxemia

**DOI:** 10.1371/journal.pone.0035066

**Published:** 2012-04-11

**Authors:** Jacob V. Stidsen, Reza Khorooshi, Martin K. U. Rahbek, Katrine L. Kirketerp-Møller, Pernille B. L. Hansen, Peter Bie, Karin Kejling, Susanne Mandrup, Samuel Hawgood, Ole Nielsen, Claus H. Nielsen, Trevor Owens, Uffe Holmskov, Grith L. Sørensen

**Affiliations:** 1 Cardiovascular and Renal Research, Institute for Molecular Medicine, University of Southern Denmark, Odense, Denmark; 2 Department of Neurobiology Research, Institute for Molecular Medicine, University of Southern Denmark, Odense, Denmark; 3 Department of Biochemistry and Molecular Biology, University of Southern Denmark, Odense, Denmark; 4 School of Medicine, University of California San Francisco, San Francisco, United States of America; 5 Department of Pathology, Odense University Hospital, Odense, Denmark; 6 Institute for Inflammation Research, Copenhagen University Hospital, Rigshospitalet, Copenhagen, Denmark; University of Padova, Italy

## Abstract

Pulmonary surfactant protein D (SP-D) is a host defence lectin of the innate immune system that enhances clearance of pathogens and modulates inflammatory responses. Recently it has been found that systemic SP-D is associated with metabolic disturbances and that SP-D deficient mice are mildly obese. However, the mechanism behind SP-D's role in energy metabolism is not known.

Here we report that SP-D deficient mice had significantly higher ad libitum energy intake compared to wild-type mice and unchanged energy expenditure. This resulted in accumulation but also redistribution of fat tissue. Blood pressure was unchanged. The change in energy intake was unrelated to the basal levels of hypothalamic Pro-opiomelanocortin (POMC) and Agouti-related peptide (AgRP) gene expression. Neither short time systemic, nor intracereberoventricular SP-D treatment altered the hypothalamic signalling or body weight accumulation.

In ad libitum fed animals, serum leptin, insulin, and glucose were significantly increased in mice deficient in SP-D, and indicative of insulin resistance. However, restricted diets eliminated all metabolic differences except the distribution of body fat. SP-D deficiency was further associated with elevated levels of systemic bacterial lipopolysaccharide.

In conclusion, our findings suggest that lack of SP-D mediates modulation of food intake not directly involving hypothalamic regulatory pathways. The resulting accumulation of adipose tissue was associated with insulin resistance. The data suggest SP-D as a regulator of energy intake and body composition and an inhibitor of metabolic endotoxemia. SP-D may play a causal role at the crossroads of inflammation, obesity, and insulin resistance.

## Introduction

SP-D is an innate host defense lectin and widely used as seromarker of pulmonary integrity [1]. SP-D can be measured in serum by specific assays [2], [3] and appears to be increased early in the course of lung injury, and the elevated concentrations are mainly thought to reflect pulmonary epithelial injury [1], [4]. SP-D synthesis is located to the alveolar lining, but in addition the protein is expressed in endothelial cells, various epithelial linings including the gastrointestinal tract and the brain [5], [6], [7].

Systemic SP-D was recently demonstrated to be decreased in type II diabetes, positively associated with insulin sensitivity after oral glucose tolerance test [8] and negatively associated with obesity [9], [10]. In contrast, high serum SP-D levels were recently associated with cardiovascular disease (CVD) and total mortality in patients with documented coronary artery disease, independent of other well-established risk factors eg. age, sex, and cigarette smoking. SP-D was reported more discriminative of CVD and total mortality than any of these factors except for age [11].

The nature of the relation between SP-D and metabolic disturbances is unknown. Abnormalities in pulmonary function are considered as risk factors for development of type 2 diabetes [12]. In addition, inflammatory responses in the lung influence adipocyte production of leptin and adiponectin. On the other hand, overweight and obesity are considered to be significant risk factors for pulmonary disease and respiratory symptoms [13], [14]. The underlying mechanisms whereby the pulmonary system may affect metabolism are diverse [13] and involve altered levels of several innate immune receptors [15] besides SP-D, but exact direction of the association between reduced lung function and metabolism, as well as the pathophysiological mechanisms, have not been established.

SP-D's role in innate immunity is to enhance phagocytosis of microorganisms [16] and to directly inhibit growth of pathogens by increasing membrane permeability [17]. SP-D is further known to bind gram-negative bacterial lipopolysaccharide directly and may thus take part in the scavenging of the endotoxin [18]. SP-D also down-regulates bacterial endotoxin-elicited inflammatory responses in alveolar macrophages by high-affinity binding to the Toll-like receptor 4 (TLR4) and MD-2 complex [19]. The importance of SP-D in antimicrobial and inflammatory responses has been determined using Spd−/− mice [20].

Unchallenged Spd−/− mice develop pulmonary emphysema and alveolar phospholipid accumulation at a young age in addition to chronic pulmonary inflammation associated with increased metalloproteinase activity, NF-κB activation, and macrophage oxidant production [21], [22]. In humans and mice, the presence of pulmonary emphysema is usually associated with progressive loss of tissue involving fat mass and fat-free mass, respiratory cachexia secondary to elevation of inflammatory cytokine levels, and the reduction of anabolic hormone levels [23], [24], [25]. However, despite the deranged pulmonary phenotype, the SP-D deficient mice appear mildly obese [9]. Systemic lipid homeostasis is also influenced by SP-D deficiency, as it causes elevated plasma triglycerides and high-density lipoprotein cholesterol (HDL-C); diet-induced formation of atherosclerotic lesions is depressed in Spd−/− mice [26]. Recently, it was demonstrated that SP-D expression is significantly increased in pancreatic β-cells under conditions of pregnancy-induced insulin resistance and in newly formed β-cells making the molecule a specific marker for new insulin producing cells [27], [28]. Together these observations suggest that SP-D may have functions, which affect the energy metabolism in addition to the well-known immune functions.

Obesity and metabolic disturbances may be evoked by or influenced by a multitude of different stimuli eg. energy intake, energy expenditure, central appetite regulation, modulation of adipocyte function, and metabolic endotoxemia. Energy intake is a major contributor and is regulated by a complex network involving hypothalamic as well as extrahypothalamic structures in the brain. The neuronal networks are associated with both orexigenic and anorexigenic signals, of which POMC and AGRP play central roles in the hypothalamic regulation of appetite and food intake [29]. Extrahypothalamic pathways involved in the regulation of energy intake, hedonic eating, and the role of leptin herein are recognized, but not well described [30]. Adipose tissue is dynamically linked with the immune system; indeed, the activity of macrophage cells has a key role in adipocyte function and increased abundance of macrophages is found in adipose tissue [31], [32]. Adipose tissue macrophages are thus suggested to be an important factor for systemic insulin sensitivity [33]. Obese and diabetic mice have been reported to display enhanced intestinal permeability, which may result in increased systemic endotoxin derived from gut microbiota. On the other hand, bacterial endotoxin may trigger and maintain a low-tone continuous inflammatory state and in this way metabolic endotoxemia has been suggested to evoke obesity and insulin resistance in mice [34].

In addition to the pulmonary epithelium, other tissues have been found to synthesize SP-D, notably the brain, metabolic organs like the insulin producing pancreas, the gut, and the endothelium throughout the body. The widespread distribution justifies speculations about the ways by which SP-D may affect metabolism. The present study set out to explore a set of different metabolic variables, which could be affected by SP-D deficiency and cause mild obesity. Initially, the body mass development of the SP-D deficient mouse was compared to the corresponding wild-type body mass during ad libitum energy intakes and fixed energy intakes. In addition, the effects of recombinant SP-D administration on c-fos and neuropeptide gene expression within hypothalamus and body weight were investigated. We further performed measurements of body composition, blood glucose, insulin and leptin, measured blood pressure, estimated the adipocyte and macrophage composition in fad pads, and measured basal systemic endotoxin levels. The variables were analyzed in the SP-D deficient mice in order to identify metabolism-related pathways involved in the overall changes in body weight and body composition. The major findings from the investigations include hyperphagia, redistribution of body fat, insulin resistance, and increased systemic bacterial endotoxin levels, indicative of propensity for metabolic endotoxemia associated to SP-D deficiency.

## Materials and Methods

### Longitudinal studies of body weight

SP-D deficient mice (Spd−/−) on a CD-1 background were backcrossed in excess of 12 times with the C57BL/6N strain (Charles River Laboratories, Sulzfeld, Germany). Male Spd−/− and wild-type mice were fed ad libitum or with a fixed energy value of 80% or 95% of wildtype ad libitum energy intake. Mice were fed with a low fat diet (16.50 kJ/g; 59.3% (w/w) carbohydrate; 16.5% protein; 7% fat; (AIN-93G (829051); Special Diets Services, Witham, Essex, UK) or a high fat diet (19.90 KJ/g; 39.0% carbohydrate; 19.7% protein; 23.1% fat; (824116; Special Diets Services, Witham, Essex, UK)). The two diets were isoenergetically exchanged. This means that the composition of all ingredients (vitamins and protein etc.) were equal per energy unit, except from corn starch and fat. Diets were provided as powder in a stainless steel feeder to minimize spillage. Mice were housed individually in macrolon type II cages, except that mice fed 95% of normal energy intake were housed 6 to a macrolon type III cage. Cages contained aspen woodchip bedding (Tapvei, Brogaarden, Gentofte, Denmark) and nesting material (EnviroDri, Brogarden, Gentofte, Denmark). The environment was controlled with respect to temperature (21–24°C) and illumination (12 hour light/dark cycle with a 30 min sunset and dawn function). All mice had free access to water. Dietary treatment was initiated at 7 weeks of age. Body mass was recorded regularly and ad libitum energy intake was obtained over a period of 18 weeks. Venous blood samples were collected by puncture of the retroorbital plexus before initiation of the experiment and at the 9^th^ and 18^th^ week of diet treatment. Sampling was preceded by a 6 hour fast from 0700 to 1300. At experiment termination the mice were sacrificed by CO_2_ asphyxiation. The gonadal, retroperitoneal, and subcutaneous inguinal fat pads were subsequently dissected and weighed. All animal experimental protocols were performed under a license obtained from The national Animal Experiments Inspectorate who also approved the study.

### Serum glucose and free fatty acids

Serum glucose and free fatty acids were measured using a Cobas Mira analyzer (Roche diagnostics). Glucose was analyzed using the ABX Pentra Glucose HK CP kit (ABX diagnostics, Montpellier, France), and serum free fatty acids were analyzed with the NEFA-C kit (Wako Chemicals GmbH, Neuss, Germany). Assays were performed according to manufactures directions.

### Measurement of adiponection, insulin and leptin

A Luminex 100 IS (Luminex corp., Austin, TX, USA) was employed for measurement of adiponectin using the mouse single plex adiponectin lincoplex kit (MADPK-71K-ADPN, Linco Research Inc, St. Charles, Missouri, USA), and of insulin and leptin using the mouse serum adipokine lincoplex kit (MADPK-71K, Linco Research, St. Charles, Missouri, USA). More than 50 events were acquired per bead set. Assays were performed according to manufactures directions. Data analysis was performed using the StarStation ver. 2.0 software (Applied Cytometry Systems, Sheffield, UK).

### Measurements of energy, protein, and fat contents

Urine and faeces were collected over a four day period in metabolic cages concomitant with recording of water and food consumption. Urine was collected in 300 µL 1 M sulphuric acid, while residual urine on the collective tract was collected with 1% citric acid. Weight of urine, collected citric acid and faeces was recorded and frozen at −20°C for subsequent analysis.

Total nitrogen content in urine, carcass and diets was analyzed by the Kjeldahl method. Nitrogen release was facilitated by destruction at 420°C in phosphoric acid and with cupper sulphate as a catalyst (Kjeltab CK, Foss A/S, Hillerød, Denmark). Released nitrogen was extracted using the Kjeltec auto distillation unit (Foss A/S, Hillerød, Denmark). Nitrogen content was determined by titration with HCl. Protein content was estimated from nitrogen content by the conversion factor 6.25.

Energy content of urine, faeces and diets were determined using the IKA calorimeter C 5000 Control (Ika Werke GmbH & Co. KG, Staufen, Germany) by combustion in 100% oxygen at a pressure of 30 bar. Samples were homogenized and dried in a vacuum oven at 120°C before analysis.

Fat content of carcasses and diets were determined by liberation of chemically bound fatty acids by HCl hydrolysis using the hydrolysis unit SoxCap 2047 (Foss A/S, Hillerød, Denmark), followed by ether-extraction using the Soxtec2050 extraction unit (Foss A/S, Hillerød Denmark). The extracted lipid content was dried and weighed.

Assimilated energy intake was determined as gross energy intake subtracted by energy lost in faeces, and metabolizable energy intake was determined as gross energy intake subtracted by energy lost in faeces and urine.

### Indirect calorimetry

The gas exchange was measured in an open-air-circuit respiration unit (Micro-Oxymax calorimeter, Columbus Instruments, Columbus, Ohio, USA). The unit contained four small chambers with a volume of 2.0 L, in which the temperature was kept approximately constant at 22–24°C. The oxygen consumption was measured according to the paramagnetic principle and the carbon dioxide production in accordance with the infrared principle. Prior to each measurement, the gas sensors were calibrated against a gas mixture of known concentration and outdoor air.

Animals were placed individually in respiration chambers and gas exchange was measured for 22 hours at an inflow of ambient air into the chambers of 0.3 L/min. Ambient air and mixed cage air were sampled for 1 min, both preceded by 4 min of purging to allow sensors to adjust, for a total time of 10 min per chamber. The actual data from each chamber were recalculated to standard conditions (STP: temperature 0°C, pressure 760 mm Hg). Body core temperature was measured using a rectal probe prior to gas exchange measurements.

Energy expenditure (EE) was calculated from O_2_ consumption, CO_2_ production and nitrogen content in urine (UN) in accordance with Brouwer [35] and oxidation of carbohydrate (OXCHO) and fat (OXF) [36].

EE, kJ = 16.18×O_2_, ml+5.02×CO_2_, ml−5.99×UN, mg

OXCHO, kJ = (−2.968×O_2_, ml+4.174×CO_2_, ml −2.446×UN, mg)×17.58

OXF, kJ = (1.719×O_2_, ml−1.719×CO_2_, ml−1.963×UN, mg)×39.76

### Measurements of blood pressure in conscious mice

Male and female Spd−/− and wild-type mice 8–9 months of age were used for invasive measurements of mean arterial pressure (MAP). Male Spd−/− and wild-type mice 10–11 months of age were used for MAP measurements after N^G^-nitro-L-arginine methyl ester (L-NAME) infusion (n  =  4–6 per treatment group). Animals were anaesthetized with ketamine hydrochloride and xylazine (50 mg/kg and 10 mg/kg, IP). The anaesthesized mice were pinched between the toes in order to check for reflexes before starting the operational procedure. Catheters were placed in the left femoral artery and vein, tunnelled subcutaneously to the back of the neck and attached to a swivel, as described previously [37]. The mice were allowed to recover for 3 days before MAP was measured by a pressure transducer (Föhr Medical Instruments GMBH, Germany) connected to an amplifier (Electronic shop, Institute of Medical Biology, University of Southern Denmark). The signal was digitized by an analog-to-digital converter (USB 6008, National Instruments, Austin, TX, USA), and recorded using customized Lab View software (National Instruments). MAP was measured for 1 hour before L-NAME infusion, and after 24 hour intravenous L-NAME infusion (8.6 mg·kg^−1^·day^−1^).

### Administration of exogenous SP-D

The effects of exogenous SP-D treatment on body weight, food intake, and neuropeptide gene expression profile in the hypothalamus were tested in male Spd−/− and wild-type mice (8 weeks old) by injection of native SP-D or saline either intravenously (iv) or intracerebroventricularly (icv), or by infusion of recombinant SP-D subcutaneously by osmotic minipumps.

For iv or icv treatments, 8 weeks old SP-D deficient and wild-type male mice were used (n = 5–6 pr treatment group). After two-three days of baseline measurements with free access to standard rodent chow (Altromin 1320, Altromin, Lage, Germany) the tail vein was injected with saline (200 µl) or 100 µg/ml human SP-D purified from late amniotic fluid as described previously [38]. Mice were weighed and their food intake was measured two-three days prior to treatments in order to achieve a baseline food intake and bodyweight. All animals had free access to standard rodent chow. In one group of mice, single injection was followed by measurement of neuropeptide expression 2 h later. In another group, 3 injections were given with 2 day intervals to investigate changes in body weight.

Icv administration was performed by placing anesthesized mice in a stereotactic instrument (KOPF model 900 with model 5000 microinjection unit attached, and with a Hamilton syringe 75RN, 5 µL and a GA26S cannula). Anaesthesia was performed as described for measurements of blood pressure, except that Ketamine/Xylazine concentrations were raised to 125 mg/kg and 25 mg/kg, respectively. Saline (2 µl) or 2 µl of human SP-D in solution (see above) were injected at the coordinates 0.4 mm caudal to bregma, 1 mm lateral to the midline on the right side, at depth 2 mm [39]. In two groups of mice, neuropeptide expression was quantified after 45 minutes and 2 hours, respectively. In a third group, food intakes and body weights were measured for three days after one icv injection. All experiments included sham operated, untreated animals. The anaesthesized mice were pinched between the toes in order to check for reflexes before starting the operational procedure.

Before histological analysis, iv or icv treated mice were euthanized with Pentobarbital and perfused with ice cold PBS, the brains were removed, the hypothalami were dissected out, placed in cryo tubes, snap frozen, and subsequently stored at −80°C.

Subcutaneous infusion of 0.82 mg/ml recombinant full length SP-D [18] or saline was performed using Alzet osmotic minipumps (1007D, Scanbur, Karlslunde, Denmark) placed subcutanously under isoflurane anaesthesia in 14 weeks old male Spd−/− mice (n = 4 per treatment group). Body weight and food intake were measured daily for 3 days prior to and for 6 days during infusion. The human recombinant SP-D was measured in the terminal blood sample using ELISA as previously described [3]. A test for biological activity of the recombinant SP-D was performed by detection of SP-D induced phosphorylation of p38 as previously described [40] using human endothelial cells (HPMEC-ST1.6R kindly provided by Dr. Ronald Unger, Institute of Pathology, Johannes Gutenberg University, Mainz, Germany).

### Sample preparation for RT-PCR

Total RNA was purified using TRIzol RNA isolation reagent (Invitrogen Life Technologies) according to the manufacturer's protocol. One µg of RNA from each sample was incubated with Moloney murine leukemia virus RT (Invitrogen Life Technologies) according to the manufacturer's protocol, using random hexamer primers.

PCR reaction and real-time detection of the PCR product accumulation were performed using ABI Prism 7300 Sequence Detection Systems (Applied Biosystems, Foster City, CA). The qPCR was performed for POMC (Forward; TGCTTCAGACCTCCATAGATGTGT, Reverse; GCGAGAGGTCGAGTTTGCA, Probe; CTGGCTTGCATCCG), AGRP (Forward; GCTCCACTGAAGGGCATCA, Reverse; TGTCTTCTTGAGGCCATTCAGA, Probe; CAGAGTTCCCAGGTCT) and c-fos (Forward; GCATGGGCTCTCCTGTCAA, Reverse; GGCACTAGAGACGGACAGATC, Probe; ACACAGGACTTTTGC).

The ribosomal 18S rRNA (Applied Biosystems) was chosen as endogenous control. Each reaction was performed with TaqMan 2x Universal PCR Master Mix (Applied Biosystems), primer and TaqMan probe, and sterile milliQ water. Relative expression values were then calculated by dividing the expression level of the target gene by the expression level of 18S rRNA, as described previously [41].

### Stereological estimation of adipose tissue macrophages, adipocytes and adipocyte size

Six-week-old SP-D deficient male mice and wild-type male mice fed with 95% energy restriction were used for the experiment (n = 3). After termination, the right gonadal, retroperitoneal, and subcutaneous inguinal fat pads were fixed in 4% formaldehyde and tissue sections were embedded in paraffin. Macrophages were stained with F4/80 rat anti-mouse antibody (MCAP497, Serotech, Oxford, UK) and counter stained with conventional Mayers Hematoxylin and eosin stain. The numbers of adipocytes and macrophages in three dimensions were determined using conventional stereology (10 randomly selected sections from each sample and reference tissue and counting frames 1000 µm apart [42], [43]). Adipocyte volumes were estimated using the point-sampled linear intercepts principle [42]. All stereological procedures were performed using an Olympus BH-2 light-microscope fitted with an Olympus digital camera, CAST2 software (Olympus, Denmark), and a MW multicontrol 2000 specimen stage connected to the computer.

### Measurement of basal levels of systemic endotoxin

Eighteen weeks old male mice deficient for SP-D and wild-type male mice fed a ground standard chow (Altromin 1320, Altromin, Lage, Germany) were used for the experiment (n = 15–16). Mice were fasted for the last four hours during the dark phase. Following the light was switched on and the mice were given access to the chow for two hours before blood samples were drawn. Sera were obtained by retro-orbital puncture and collected using sterile glass capillaries into glass vials, which were heated for 30 minutes at 250°C before use. Sera were subsequently diluted 1/40 in Pyrosperse (Lonza Copenhagen Aps, Vallensbæk Strand, Denmark). Gram-negative bacterial endotoxin levels were measured using the Q CL-1000 LAL-assay (Lonza Copenhagen Aps, Vallensbæk Strand, Denmark) according to the manufacturer's instructions.

### Statistical analysis

Comparisons between experimental group means were evaluated by means of two-way ANOVA, with diet and genotype as variables. Differences between the two genotypes in their response to diet type were evaluated with the interaction term in the ANOVA model, genotype*diet, when relevant. When the genotype effect was unaffected by diet, data was presented as least square means for the two genotypes, disregarding the type of diet. T-test was used as post hoc test. Data used for multiple regression modelling and matching ANOVA were normally distributed. Residuals from the regression models were also normally distributed as well as uniform. The Kruskal-Wallis with Dunns post hoc test was applied for non-parametric data. P-values<0.05 were considered significant. Statistical analyses and graph presentations were performed using Stata Intercooled 8.0 (www.stata.com), Microsoft Excel 2008, or GraphPad Prism 5.

## Results

### Spd−/− mice have elevated body mass

**Figure 1:**
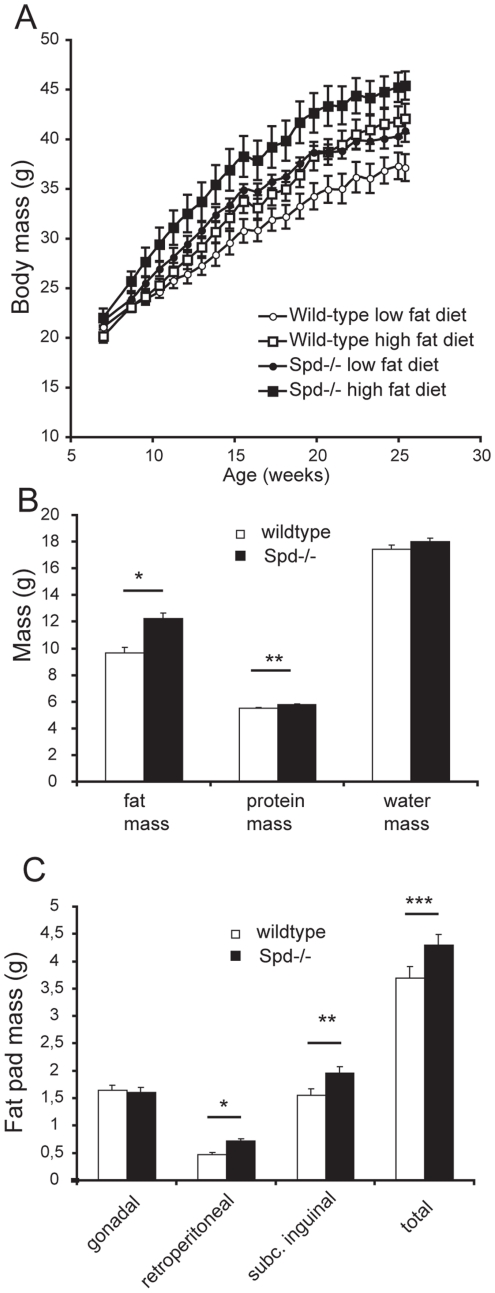
Mean body masses of male mice fed ad libitum for 18 weeks. A) Spd−/− mice had a higher body mass than their wild-type counterparts. ANOVA of AUC showed a genotype effect (p = 0.005) and a diet effect (p = 0.02). Diet did not influence the body mass difference of genotypes. Data are means ± SE, n = 6. B) Carcass mass of protein, fat and water in 28 week old mice. Protein and fat mass was increased in Spd−/− mice. Genotype effect, * p<0.001 and ** p = 0.038. Data are least square means ± SE, n = 11–12. C) Mean masses of three fat pads in 28 week old mice. The gonadal fat pad mass was similar in the two genotypes. Retroperitoneal fat, subcutaneous fat and the total of the three pads were increased in Spd−/− mice. Genotype effect, * p = 0.001, ** p = 0.03, *** p = 0.047. Data are least square means +/− SE, n = 11–12.

The body mass in 7 weeks old male Spd−/− mice was marginally increased compared to wild-type mice (21.6±0.4 vs. 20.4±0.3 g, p = 0.02, n = 24); however, following 18 weeks ad libitum feeding Spd−/− mice displayed significantly increased body masses compared to wild-type mice assessed by single measurements as well as by integrated areas under the curve (AUC) (Fig. 1A). During the course of the feeding study Spd−/− mice developed a 10% higher body mass compared to wild-type mice, evaluated as genotype AUC. The difference in body masses peaked at 14% at 19 weeks of age (39.6±1.0 vs. 34.8±1.0 g, p = 0.002, n = 12). The differences in body masses between the two genotypes were similar during high fat and low fat diets (interaction-term, diet*genotype in the ANOVA). At 28 weeks of age, total body protein content was increased by 4.8% (p = 0.038) and body fat mass was increased by 27% (p<0.001) in Spd−/− mice relative to wild-type levels (Fig. 1B). The mean masses of the gonadal fat pads were equal in the two genotypes; however, both the retroperitoneal and the subcutaneous fat pads were enlarged in the Spd−/− mice (Fig. 1C).

### Body masses in Spd−/− and wild-type mice are similar when the energy intake is identical

**Figure 2:**
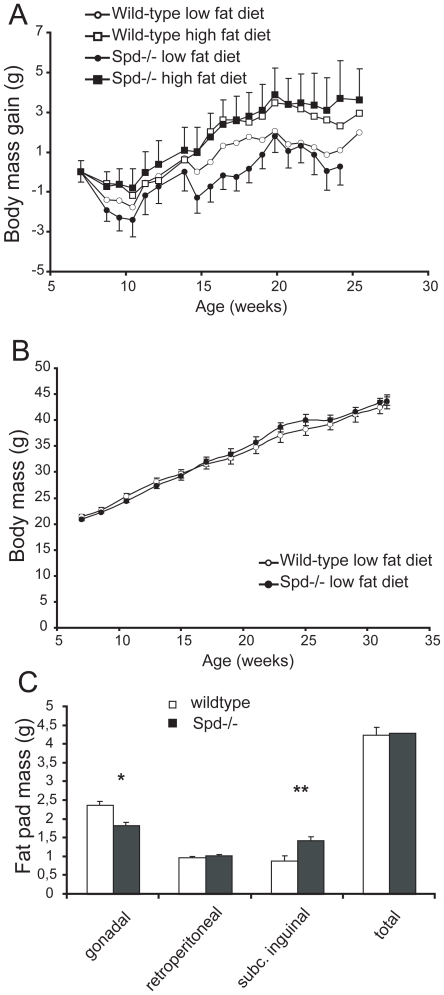
A) Mean body mass gain in male Spd−/− and wild-type mice fed on a 80% energy restricted diet for 18 weeks. ANOVA of body mass gain after 18 weeks did not show any genotype difference. B) Mean body masses of Spd−/− and wild-type mice fed a 95% restricted low fat diet. The body masses in Spd−/− and wild-type mice were equal after 25 weeks of diet treatment. C) The masses of three fat pads from 32 week old mice fed a 95% restricted low fat diet. The total mean mass of the three pads and the retroperitoneal pads was equal in the two genotypes. The gonadal fat pad mass was decreased and the subcutaneous pad was increased in Spd−/− mice. * p = 0.003, ** p = 0.008. Data are means + or − SE, n = 6.

Wild-type and Spd−/− mice were submitted to restricted feeding in order to investigate if hyperphagia contributed to the obese phenotype. When Spd−/− and wild-type mice were fed a diet providing 80% of the ad libitum wild-type energy intake, Spd−/− mice did not increase body mass compared to wild-type mice (Fig. 2A), and the body composition was also similar in the two genotypes (data not shown). The same picture was seen with mice fed a diet providing 95% of ad libitum energy intake (Fig. 2B). The retroperitoneal, gonadal and subcutaneous inguinal fat pads of Spd−/− mice and wild-type mice fed a 95% restricted low fat diet for 25 weeks were dissected and weighed. In keeping with the lack of difference in body weight, the total masses of the three fat pads were similar between genotypes (Fig. 2C). The retroperitoneal deposits did not display any significant differences in masses between the genotypes either. In contrast, the gonadal fat deposits in Spd−/− mice were 23% (p = 0.003) smaller, and the subcutaneous deposits were 61% (p = 0.008) larger than those of wild-type mice (Fig. 2C). All other organs investigated had similar masses in the two genotypes, except for a 15% increment in kidney size in the Spd−/− mice (0.52±0.02 vs. 0.45±0.01 g, p = 0.039).

### Spd−/− mice have elevated energy consumption

**Figure 3:**
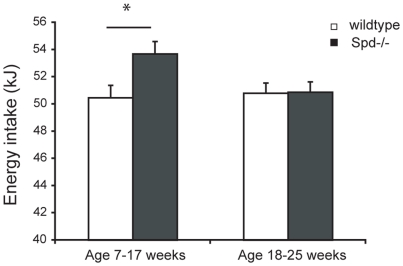
Average energy intake in Spd−/− and wild-type mice. At age 7–17 weeks energy intake was significantly higher in Spd−/− mice, but from age 18 to 25 the energy intake was identical. Two-way anova, genotype effect, * p = 0.02. Data are least square means + SE, n = 12.

Energy intake was recorded during the 18 weeks of ad libitum feeding with isoenergetically exchanged high or low fat diets. The daily energy intake was not constant throughout the period and was calculated, therefore, as the slope of the accumulated energy intake from 7–17 weeks of age and again from 18–25 weeks of age. The energy intake of Spd−/− mice was elevated by 3.2±1.3 kJ/day relative to wild-type mice (53.7±0.9 vs. 50.5±0.8 kJ/day, p = 0.02, n = 12) at 7–17 weeks of age, but showed no difference at age 18 to 25 weeks (Fig. 3), and 93% of the difference in accumulated energy intake between the genotypes was achieved during this early experimental period. The increase in energy intake was independent of diet composition (diet*genotype).

Gastrointestinal assimilation (digestion and absorption) of energy was not affected by energy intake, diet, or genotype as determined by the quotient between assimilated and gross energy intake. Neither was metabolizable energy affected by the three variables (data not shown).

### The energy expenditure is not significantly different between Spd−/− and wild-type mice

Measurements of energy expenditure (EE) and substrate oxidation were obtained in order to investigate if the observed obese phenotype in the Spd−/− mice could be partly explained by alterations in EE. EE was measured after 21 weeks in mice fed ad libitum or on an 80% restricted diet.

**Figure 4:**
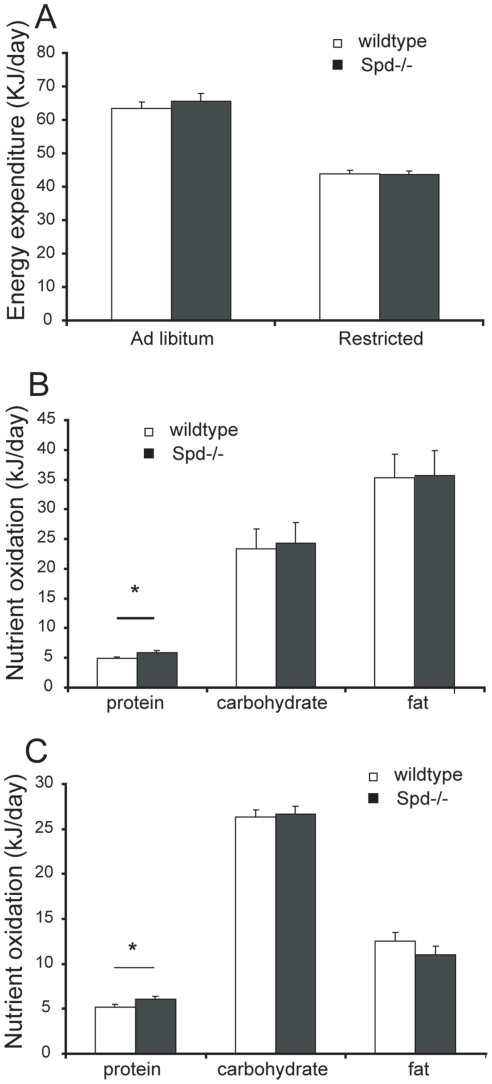
Heat exchange in 28 week old Spd−/− and wild-type mice. A) Heat exchange per animal was not significantly different between genotypes. B) Nutrient oxidation in ad libitum fed mice. Protein oxidation showed a borderline genotype effect, * p = 0.049. C) Nutrient oxidation in restricted fed mice. Protein oxidation showed a genotype effect, * p = 0.035. Data are least square means + SE, n = 11–12.

In line with the observation that bodymass differences between the genotypes disappeared with energy restricted feeding, EE was not significantly different in Spd−/− mice compared to wild-type mice fed ad libitum (Fig. 4A). After correction for differences in body mass, the EE remained similar between Spd−/− and wild-type mice when heat exchange was expressed relative to metabolic body size (kg^0.75^) as well as when expressed relative to lean body mass (data not shown). Moreover, there was no measured disturbance in core temperature measured with a rectal probe and thus no apparent disability to thermoregulate in the Spd−/− mice. Spd−/− and wild-type mice fed a diet restricted to 80% of wild-type ad libitum energy intake likewise had identical EE (data not shown). Oxidation of carbohydrate and fat was similar in the Spd−/− mice and wild-type mice, while a small difference was seen in protein oxidation in ad libitum fed mice (Fig. 4B) as well as in mice fed a 80% energy restricted diet (Fig. 4C). Moreover, oxygen consumption in young mice (8 weeks old) mice of similar body mass was not significantly different between the genotypes (data not shown).

### Fasting serum insulin, glucose and leptin are elevated in ad libitum fed Spd−/− mice

Fasting serum insulin, glucose, and leptin were measured after 18 weeks of high fat or low fat diet (Table 1). The fasting glucose concentrations measured in all ad libitum fed mice at these time points were above 10 mmol/l (180 mg/dL) indicating that they had become hyperglycemic.

**Table 1:** Insulin, glucose and leptin in Spd−/− mice and wild-type mice.

Feeding group	Insulin (ng/ml)	Glucose (mmol/l)	Insulin*Glucose ()	Leptin (ng/ml)
WT LF AL	1.80±0.45	10.4±0.50	18.20±3.88	31.17±6.80
Spd−/− LF AL	2.67±0.27	12.2±0.48*	33.05±3.86*	28.40±3.96
WT HF AL	1.78±0.32	12.04±0.64	21.06±2.88	20.67±1.26
Spd−/− HF AL	3.24±0.40*	12.4±0.51	40.95±5.94*	45.50±7.90*
WT LF R	0.47±0.04	6.86±0.16	3.22±0.28	0.67±0.07
Spd−/− LF R	0.43±0.09	6.05±0.48	2.83±0.73	1.08±0.17
WT HF R	0.49±0.02	7.52±0.44	3.71±0.29	2.02±1.00
Spd−/− HF R	0.53±0.14	7.28±0.48	2.94±0.64	2.40±0.62

Data are means ± SE. WT = wildtype mice, Spd−/− = SP-D deficient mice, LF = low fat diet, HF = high fat diet, AL = ad libitum, R = 80% energy restricted.

* p<0.05.

In the ad libitum fed Spd−/− mice the same trends were observed for measurements of glucose and insulin disregarding the diet type although significance was not observed in all groups (Table 1). Fasting serum insulin was increased by 48% (ns) and 82% (p = 0.008) when fed the low fat diet and high fat diet, respectively. Fasting serum glucose was significantly elevated by 17% (p = 0.03) in low fat diet fed Spd−/− mice compared to wild-type mice. High fasting insulin or glucose is an indicator of insulin resistance and the insulin-glucose product was increased by 82% (p = 0.02) and 94% (p = 0.01) in low fat diet and high fat diet fed Spd−/− mice, respectively (Table 1). Multiple linear regression of the insulin-glucose product with body-mass and genotype as independent variables revealed that the SP-D deficient genotype influenced the proxy for insulin resistance independently of body mass in the ad libitum fed mice (r = 0.76, body mass: p = 0.024, genotype: p = 0.006). According to the regression model, the genotype influenced the proxy by 13,100±4,300 pg*mol/l^2^, while an increase in body mass of 1 g increased the product by 1,200±500 pg*mmol/l^2^. Neither serum insulin nor serum glucose was affected by SP-D deficiency in the mice fed with 80% energy restriction.

**Figure 5:**
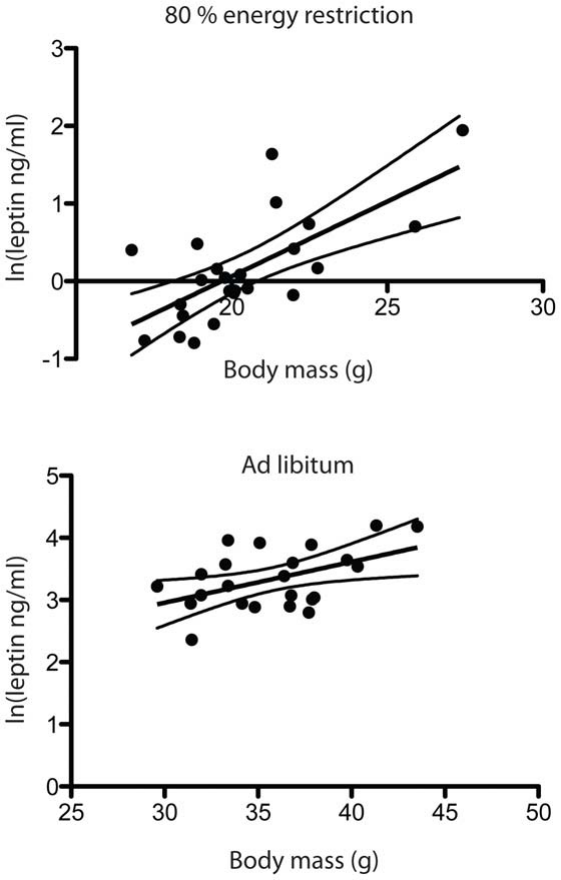
Linear association between ln(leptin) levels and mouse body weights. Mice from different feeding groups show linear relation between systemic leptin levels and body weights (Spd−/−, wild type, high fat, low fat) when fed with 80% energy restriction or ad libitum.

There was great variability in measurements of serum leptin. Fasting serum leptin was increased by 120% (p<0.05) in the ad libitum and high fat diet fed Spd−/− mice. There was no significant genotype difference in leptin levels when mice were fed with 80% energy restriction (Tab. 1). Likewise, with the 95% energy restriction there was no significant alteration in the systemic levels of leptin (data not shown). When analysed separately, leptin levels showed a significant correlation to body mass among the energy-restricted animals (R^2^ = 0.47, p = 0.0002), as well as among the ad libitum fed mice (R^2^ = 0.23, p = 0.02) (fig. 5).

Adiponectin levels appeared with increased levels in the energy restricted animals, 1,400±100 pg/ml compared to 1,100±100 pg/ml in the ad libitum fed animals (p = 0.049). Neither genotype nor diet showed any impact on adiponectin levels (data not shown).

### Mean arterial pressure in conscious mice

Mean arterial blood pressures and heart rates were measured in 8–9 months old Spd−/− mice and wildtype mice fed a standard diet ad libitum in order to test if SP-D deficiency would evoke hypertension secondary to the metabolic changes. However, there were no genotype effects in the variables. The mean arterial pressures obtained in wild-type and Spd−/− female mice were 97±10 mmHg and 98±6 mmHg, respectively. The mean arterial pressures in the corresponding male mice were 106±5 mmHg and 104±6 mmHg, respectively. Infusion of L-NAME raised arterial pressure similarly in male wild-type and Spd−/− mice (13.5±3.7% vs. 10.5±1.6%). Likewise there was no difference between heart rates in the genotypes.

### Administration of exogenous SP-D affected neither central signalling nor energy intake

In order to test if treatment with exogenous SP-D could modulate central appetite regulating pathways in the hypothalamus, we measured hypothalamic c-fos gene expression in response to different treatment regimes with native human SP-D. SP-D deficiency had no effect on the basal level of c-fos, AGRP or POMC message in the hypothalamic tissue. Likewise, analysis of c-fos, POMC, and AGRP gene expression after one dose icv or 3 doses over 6 days iv did not show any differences between treatment groups.

In accordance with the above, neither intravenous injections nor icv SP-D treatment altered energy intake in either Spd−/− mice or wild-type controls in ad libitum fed mice. Continuous administration by osmotic minipumps during 7 days altered neither bodyweight development nor food intake in the period (data not shown). At the end of the experiment, human recombinant SP-D in the mouse circulation was measured by ELISA. There was no detectable signal in the vehicle infusion group, but 48±20 ng/ml rSP-D was retrieved in the treated group.

### Cellular composition in adipose tissue

In order to test if there were apparent alterations in the cellular composition in adipose tissues, three gonadal pads from each genotype fed with 95% energy restriction were fixed for histological examination. We used mice fed with 95% energy restriction in order to investigate mice of different genotypes but with identical bodyweights closely resembling the normal wildtype weight. Infiltration of the adipose tissue with macrophages was counted by stereology; however, no difference was seen between body weight matched SP-D deficient mice and WT mice and likewise there was no difference in adipocyte size between genotypes (data not shown). Macrophage density was uncorrelated to the size of the gonadal fat pad, but it was significantly associated to total body mass (r = 0.90, p = 0.009) as were the ratio of macrophages to adipocytes (r = 0.97, p = 0.001) and total number of macrophages per fat pad (r = 0.99, p<0.001).

### Measurement of basal levels of systemic endotoxin

**Figure 6:**
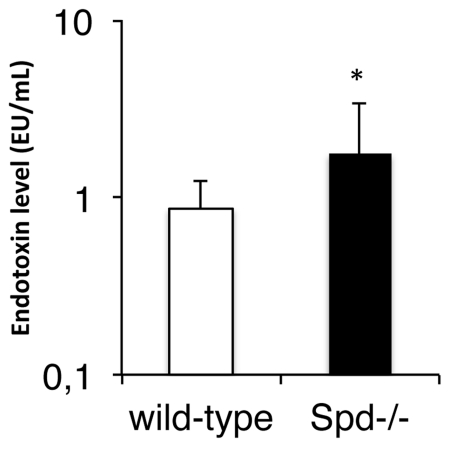
Serum endotoxin levels. Serum endotoxin levels were significantly increased in 16 SP-D deficient mice compared to 15 wild-type mice during the light phase period and fed a standard chow ad libitum. * p<0.05. Data were logaritmically transformed to obtain normality before testing. Data are means + standard deviations.

Gram negative baterial endotoxin levels were measured in mice fed a standard chow. The blood samples were drawn during the early hours of the light phase in order to avoid fluctuations reported to occur late in the dark phase [34]. An approximate 2-fold increase in the basal systemic endotoxin level was detected in the SP-D deficient mice when compared to wild-type levels in two independent experiments with one depicted in figure 6 (1.77±1.60 EU/mL versus 0.86±0.36 EU/mL, p<0.05).

## Discussion

In the present study, a mouse strain carrying a disrupted gene encoding SP-D was used to investigate the nature of SP-D modulation of body weight and metabolism. The main results derived from the explorations of the SP-D deficient phenotype include hyperphagia with no apparent effect evoked by short-term SP-D administration and no indication for involvement of hypothalamic appetite regulation. Moreover, the SP-D deficient phenotype was characterized by redistribution of body fat, insulin resistance, and increased systemic bacterial endotoxin levels, indicative of propensity for metabolic endotoxemia.

Causality between energy intake and expenditure and the obese phenotype has previously been addressed in various genetically modified strains including the extremely obese genotypes such as the CH4r−/− and the ob/ob mice [44], [45], [46]. Energy restricted Spd−/− mice did not have an increased gain of body mass, fat/lean mass ratio, or a decreased metabolic rate. Thus, the obesity seen in the Spd−/− mice is primarily dependent upon an increased energy intake and there was no apparent alteration in gastrointestinal assimilation. Moreover, in young mice, where only small and insignificant differences in body mass had developed, oxygen consumption did not differ significantly. In contrast, hyperphagia seemed to predominate in young mice as increased energy intake was evident in Spd−/− mice from 6–7 weeks of age, but normalized in >18 week old mice.

We chose to examine hypothalamic POMC and AGRP, due to their major function as part of the melanocortin system involving in appetite regulation [47], [48], [49], [50]. In our experiments no effect of the short term SP-D treatment on hypothalamic POMC and AGRP gene expression could be measured after peripheral and/or central administration. This type of negative observation is reported previously e.g. in fasted GM-CSF deficient and hyperphagic rats [48]. Likewise analysis of c-fos induction after icv or iv treatment did not show any difference between treatment groups, neither within the genotypes receiving different compounds nor between the genotypes treated with the same compound. The lack of effect of short term exogenous SP-D on c-fos expression in hypothalamus, however, leaves open the possibility that the primary action of SP-D on energy balance is located in the extrahypothalamic area. Expression of SP-D in human brain tissue has been reported [6] and immunostaining has localized SP-D to human brain endothelium (GL Sorensen and U Holmskov, unpublished) [7]. On the basis of the performed experiments, we cannot rule out that alternative routes of administration or prolonged treatment periods with exogenous SP-D would affect the energy intake.

Given that pulmonary derangement per se may be involved in the development of the metabolic syndrome, mouse strains with pulmonary phenotypes may be predisposed for an obese or diabetic phenotype. SP-D deficient mice and Granulocyte Macrophage-Colony Stimulating Factor (GM-CSF) deficient mice may represent such strains. These strains are previously reported with resemblance between emphysematic phenotypes, although the resulting pathology may be evoked through distinct mechanisms [51]. The GM-CSF deficient mice also represent a model of mild obesity. GM-CSF, however, is reported to act through receptor mediated CNS signalling [48]. The emphysematic Spd−/− phenotype was not quantified in this study, but as the mice neither had altered energy expenditure from possible failure to thrive nor emphysema related cachexia, this aspect of the SP-D deficient phenotype was apparently not involved in the induction of the metabolic phenotype.

Development of the metabolic syndrome in humans is thought promoted by high fat diet. To investigate the effect of fat on the metabolic syndrome in Spd−/− mice, we fed the mice a diet with an increased proportion of fat. The low and high fat diets used in our study were isoenergetically exchanged to evaluate the effect on body mass development without differences in protein, vitamins and minerals to influence the results. Although the body mass of mice on a high fat diet was elevated by 9% compared to mice on a low fat diet the genotype dependent increase in body weight, was not significantly different between the different diets and thus did not determine the major outcomes of the study.

Previously low serum SP-D has been demonstrated to be associated with BMI in human studies [8], [9], [10], [52]. In the setting of obesity, the ability of the adipose tissue to produce antiinflammatory mediators such as adiponectin decreases, whereas proinflammatory cytokines such as IL-6, TNF-α, and acute phase reactants, and other innate immune mediators increase [53], [54], [55]. SP-D deviates somewhat from this picture and is found to be decreased not only in subjects with obesity, but also type 2 diabetes and is negatively associated with fasting serum glucose. Moreover, SP-D is found positively associated with insulin sensitivity, suggesting that decreased SP-D expression could be behind the association of lung function with impaired insulin actions in type II diabetes [8]. In contrast, a recent study has demonstrated that high systemic SP-D is predictive for cardiovascular mortality [11] underscoring that the interpretation about causality behind the clinical associations must come from animal studies. The data presented here support that a lack of SP-D induces hyperphagia, and fat deposits, and reduces insulin sensitivity. On the other hand, previous data indicated that SP-D induced atherosclerosis [26]. Thus, the complex clinical associations are so far supported by a similarly complex SP-D deficient phenotype.

We further observed that Spd−/− mice had increased subcutaneous fat and decrements in retroperitoneal and gonadal fat fad sizes according to feeding regime. The SP-D dependent redistribution of fat deposits may be particularly interesting in the context of atheromata formation with visceral fat being associated to disease [56], [57], [58]. In the present investigations adipocyte number, size, or the fraction of adipose tissue macrophages did not indicate that SP-D deficiency would affect the inflammatory state of the fat tissue. Moreover, no alterations in mean arterial pressure or in endothelial function assessed by the responsiveness towards L-name infusion were found to potentially explain the role of SP-D in atheroma formation.

A possible role for the gut microbiota in obesity and additional aspects of the metabolic syndrome was recently proposed [59]. In both humans and mice, the development of obesity was dependent on the relative abundance of specific bacterial phyla in the gut [60], [61], [62], and transfer of the gut microbiota from obese mice to germ-free wild-type mice led to an increase in fat mass in the recipients indicating increased capacity for host energy harvest induced by the microbiome [63]. A recently appreciated characteristic of obesity is an aberrant intestinal microbiota composition in obese individuals or animals, which appears to be linked to the obese state itself and susceptible to dietary modulation. However, whether this aberrant microbiota composition plays an etiological role in obesity or is a consequence of the diet in obesity remains to be determined as reviewed by Conterno L., et al., 2011 [64].

Lately the involvement of innate immune receptors was documented. Toll-like receptor 5 (TLR5) is expressed in the gut mucosa and it was demonstrated that TLR5−/− mice exhibited hyperphagia and developed metabolic disturbances, including insulin resistance, and increased adiposity. The changes correlated with changes in the composition of the gut microbiota [65]. Likewise, mice with loss-of-function mutation in TLR4 are protected against the development of diet-induced obesity and insulin resistance [66], [67]. It is further demonstrated that high fat diets could induce metabolic endotoxemia, which can dysregulate the inflammatory tone and trigger body weight gain and diabetes [34].

Whereas SP-D is present in the human circulation in relatively high levels [52] there is no or very low measurable SP-D in the normal mouse blood, but in this species, age or pulmonary inflammation will induce serum SP-D to measurable levels [2], [68]. SP-D is expressed in the gut epithelium [6]. SP-D may thus contribute to scavenging or neutralizing systemic endotoxin derived from gut microbiota at these sites. SP-D has been described to bind to a variety of endotoxin subtypes and to interact directly with the TLR4/CD14 complex affecting endotoxin signalling [19], [69], [70]. In this context, systemic endotoxin levels were tested in SP-D deficient mice and found significantly increased. A potentially altered composition of gut microbiata in the SP-D deficient mice remains to be explored as a consequence of prolonged SP-D treatment and of obesity per se. Nevertheless, the present data indicate that intestinal or systemic SP-D neutralization of metabolic endotoxemia may contribute to the hyperphagic and metabolic phenotype in the SP-D deficient mice.

In conclusion, we have shown that SP-D modulates energy intake, and that hyperphagia is the primary cause of the obese phenotype of SP-D deficient mice. Moreover, we have found that Spd−/− mice have altered fat distribution, a characteristic not dependent on hyperphagia, that increased insulin resistance and hyperleptinaemia were developed secondary to hyperphagia in Spd−/− mice, and that low-grade endotoxemia may contribute to the metabolic phenotype. The results are in line with association between low serum SP-D and BMI and type II diabetes in clinical stuides. The present experiments did not provide definitive mechanistic insight into the hyperphagic phenotype of the KO mice. However, SP-D mediated clearance or neutralization of endotoxin or SP-D dependent signaling in relation to hormonal control of energy intake may directly drive hyperphagia and other mechanisms of the metabolic syndrome. Further studies are needed in order to clarify the mode of action of SP-D in this context.
